# Complete Implant Wrapping with Porcine-Derived Acellular Dermal Matrix for the Treatment of Capsular Contracture in Breast Reconstruction: A Case–Control Study

**DOI:** 10.1007/s00266-022-02826-6

**Published:** 2022-03-29

**Authors:** Franco Bassetto, Laura Pandis, Gian Paolo Azzena, Eleonora De Antoni, Alberto Crema, Leonardo Scortecci, Tito Brambullo, Chiara Pavan, Massimo Marini, Federico Facchin, Vincenzo Vindigni

**Affiliations:** 1 Plastic and Reconstructive Surgery Unit, Neuroscience Department, University of Padua, Via Giustiniani 2, 35128 Padua, Italy; 2 Psychiatric Clinic, University of Padua, Via Giustiniani 2, 35128 Padua, Italy; 3 Plastic Surgery Unit, San Bortolo Hospital, Vicenza, Viale Rodolfi 37, 36100 Vicenza, Italy

**Keywords:** Capsular contracture, Breast reconstruction, Breast implant, Acellular dermal matrix

## Abstract

**Background:**

Capsular contracture (CC) represents one of the most common complications in breast reconstruction surgery, impairing final result and patients’ well-being. The role of acellular dermal matrixes (ADM) has been widely described for the treatment and prevention of contracture. The aim of the study was to evaluate the efficacy and safety of complete implant coverage with porcine-derived ADM in preventing CC limiting complications. In addition, patients’ reported outcomes were evaluated in order to define the role of ADM in improving sexual, physical and psychosocial well-being and satisfaction.

**Methods:**

42 patients who underwent surgical treatment of 46 contracted reconstructed breasts from May 2018th to May 2019th were collected in the two groups (ADM group vs. Control group).

**Results:**

The ADM group showed lower rate of CC recurrence and a higher rate of implant losses and minor complications. A significant difference was observed in red breast syndrome (27.3% in the ADM group vs. absent in control the group) and skin ulceration rates (18.2% in the ADM group vs. 4.18% in the control group). As for patients’ perceived outcomes, the ADM group showed a statistically significant higher postoperative Satisfaction of Breast Scale score compared to the control group. In addition, a significant difference was observed in the improvement of Physical Well-Being of the Chest Scale and the Satisfaction of Breast Scale after surgery, in favor to the ADM group.

**Conclusion:**

Complete implant coverage with ADM may reduce the risk of CC recurrence in breast reconstruction. An accurate patient selection allows minimizing complications improving patient well-being and satisfaction.

**Level of Evidence IV:**

This journal requires that authors assign a level of evidence to each article. For a full description of these Evidence-Based Medicine ratings, please refer to the Table of Contents or the online Instructions to Authors www.springer.com/00266.

## Introduction

Implant-based breast reconstruction is the most applied reconstructive option after mastectomy in the USA and Europe [1, 2]. Capsular contracture (CC) is a clinical condition that typically affects breast-implanted patients in which an excessive fibrous tissue formed around prostheses causes implant firmness, deformation, dislocation, patient discomfort, and pain with adverse impact on patients’ quality of life and psychological well-being. Baker classified it in four clinical grades [3, 4]. Most patients usually complain about symptoms in the first 12–24 months after primary surgery even if there is a lack of agreement regarding the time of CC development [5, 6].

CC still represents one of the most challenging complications of breast reconstruction affecting 13.7 to 45% of patients, and it is the most common reason of re-operation [7]. However, it is considered a multifactorial event depending on patient/surgery/implant-based risk factors with unknown etiology [3, 8, 9].

Clinical conditions associated with CC are subclinical infections or bacterial contamination/biofilm, smooth silicone implants, subglandular breast position, seroma, hematoma and radiotherapy (RT) [10–14].

Surgical intervention is usually indicated for the treatment of grades III and IV CC according to Baker’s classification, respectively, characterized by moderate, palpable and visible prosthesis or severe, hard, painful contraction with prosthesis distortion [15].

The best approach to reduce the risk of recurrence is still a matter of debate. Polyurethane devices [16], implants substitution using smaller size [15], the association of fat grafting, precaution in preventing the contact between the skin and the prosthesis, anterior capsulectomy (as described by Ganon et al. and Lam et al. [15, 17]) and implants coverage with acellular dermal matrices (ADM) [18, 19] have been associated with reduced risk of recurrence.

Previous clinical and animal studies support the role of ADMs in limiting the development of CC both in irradiated and non-irradiated tissues [20–27].

The use of ADMs for the treatment of capsular contracture is gaining popularity, with many papers describing the use of human-derived ADM with complete or partial implant coverage [28]. Cheng et al. in 2013 reported a novel technique with improved results in reducing capsular contracture [18]. Among many ADMs available in the market, not all of them are available worldwide. In particular, the use of human-derived ADM is limited by law in some countries. Therefore, porcine-derived ADM can be used.

We aimed in evaluating the role of complete implant wrapping with porcine-derived ADM in reducing capsular contraction recurrence and improving patients’ reported outcomes with validated Breast Q questionnaire [29].

## Materials and Methods

### Patients and Methods

A retrospective case–control study was conducted in accordance with the World Medical Association Declaration of Helsinki (June 1964) and subsequent amendments.

We performed a case–control study with 42 breast reconstruction patients affected by capsular contracture grades 3 and 4 according to Baker’s classification who underwent a revision surgery to treat capsular contracture at the Division of Plastic Surgery, at our University-Hospital, from May 2018th to May 2019th. Institutional review board approval was granted for this study.

#### Inclusion Criteria

- Patients previously treated for breast cancer with mastectomy and reconstruction with submuscular breast implant with complete implant coverage with muscle.
- Patients who underwent breast implant change for Baker grade III or IV CC from May 2018th to May 2019th.

#### Exclusion Criteria

- Patients with known hypersensitivity to or who deny porcine materials
- Breast cancer recurrence
- Chronic seroma
- Previous lipofilling
- Polyurethane covered implants

**Fig. 1:**
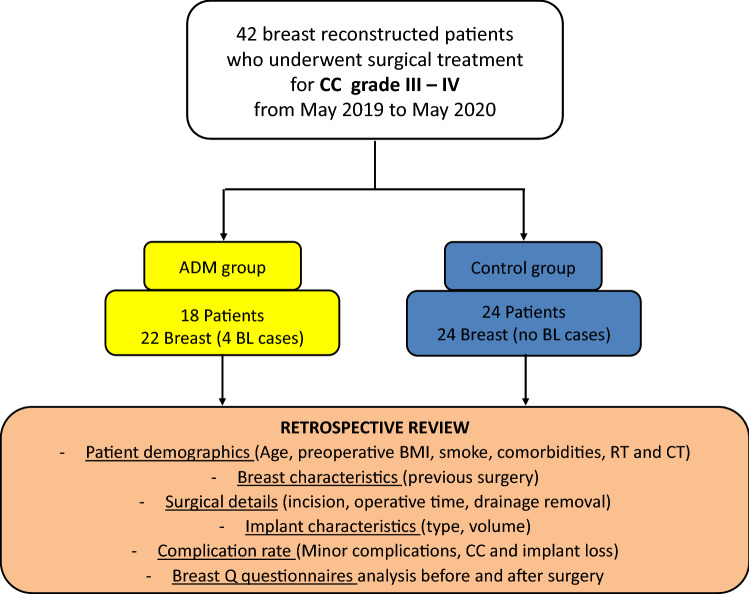
Study design. (CC = capsular contracture, ADM = acellular dermal matrix, BL = bilateral, BMI = body mass index, RT = radiotherapy, CT = chemotherapy)

24 contracted breasts (in 24 patients affected by CC) were treated with implant removal, anterior capsulectomy and implant exchange (*control group*). 22 contracted breasts (in 18 patients affected by CC) were treated with implant removal, anterior capsulectomy and implant exchange with complete breast coverage with Braxon® ADM (ADM group) (Fig. 1).

## Surgical Technique and Peri-operative Indications

The breast implant exchange was performed with complete anterior capsulectomy without plane change. The final implant size was selected after pocket measurement. Patient received prophylactic peri-operative intravenous antibiotics in the form of cefazolin, or clindamycin in case of allergy. If indicated, simultaneous contralateral surgery was performed with symmetrization or with bilateral CC treatment.

**Fig. 2:**
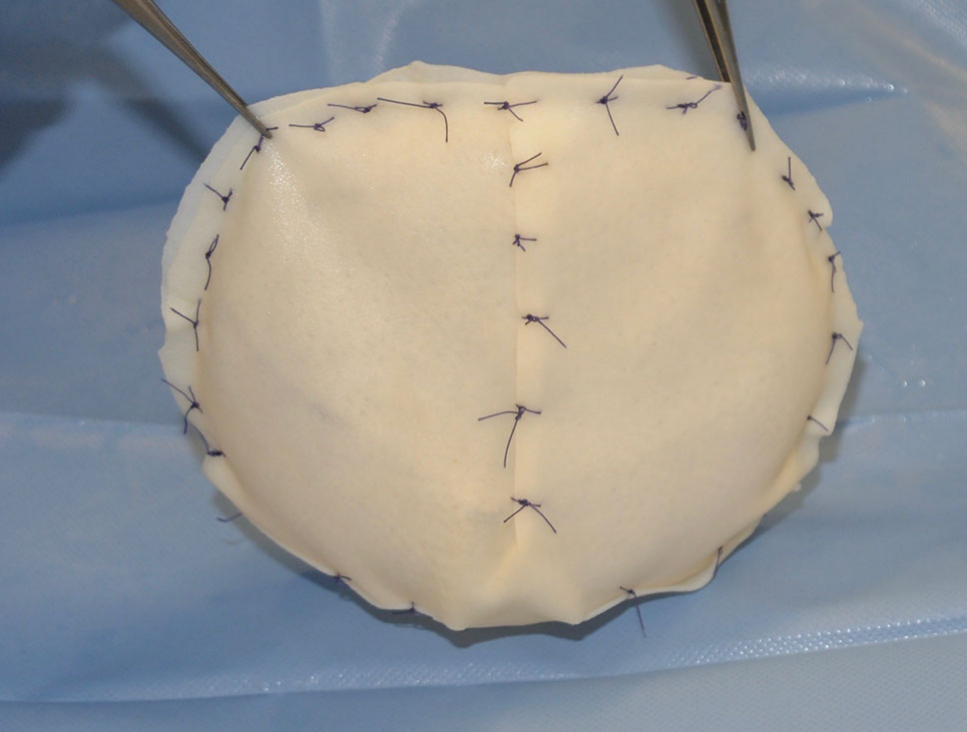
Intraoperative image of Braxon® ADM wrapped around the prosthesis

In the control group were collected patients undergoing only breast implant exchange while in the ADM group were collected patients in which a 0.6 mm porcine patented-shaped ADM Braxon® (Decomed S.r.l) was wrapped all around the prosthesis to achieve a total implant coverage after ADM hydration (Fig. 2).

**Fig. 3:**
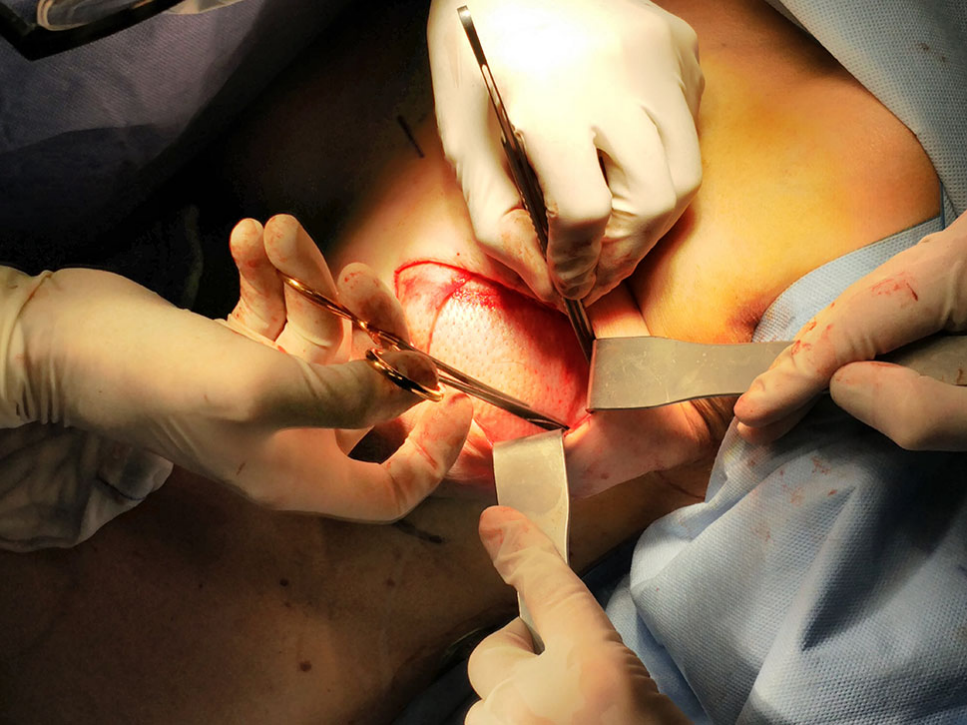
Insetting of the prosthesis covered with Braxon® ADM

The prostheses covered with ADM were then inserted in the pocket and sutured to the thoracic fascia with Vicryl™ 2-0 (Ethicon Inc., the USA), without glue (Fig. 3). In case of bilateral CC, the same surgical solution with or without ADM was applied for both breasts.

Patients of both groups were discharged the day after surgery. Post operative oral antibiotic prophylaxis was continued until drainage removal (output < 30 mL in a 24-hour period). Post-operative bra was recommended for 3 months.

## Clinical Data and Follow-Up

Age, preoperative BMI, smoke, comorbidities, history of RT and chemotherapy as well as implant volume and type, operative time were collected retrospectively from patients’ chart.

In our Unit, all patients are followed up with periodic controls during the first month and at 3, 6, 12, 18, 24 months after surgery, and Breast-Q Questionnaires (BQ) are administrated.

Post-operative complications were divided in minor complications, implant loss and CC relapse. Minor complications considered were ulcerations/necrosis not requiring revision, fever, infection, edema, hematoma not requiring re-operation, type IV delayed hypersensitivity reactions (red breast syndrome). CC was diagnosed in patients who developed a Baker III or IV grade.

Data on patients’ demographics and history, breast characteristics, surgery, implant specificity, recovery time and complications were analyzed and compared in the 2 groups in order to find potential risk factors for capsular contracture recurrence and/or implant loss.

Patients’ perceived outcomes were compared based on the data collected from the registered-trademark BREAST-Q Reconstructive Module, which includes multiple scales concerning specific aspects of Patient Satisfaction and Health-related Quality of Life and that has a specific approved Italian translation.

Breast *Q* values before and after surgery were compared within and between the two groups using the paired *t* test and the ANOVA test, respectively.

### Statistical Analysis

Statistical analysis was carried out using Microsoft® Excel® software (version 16.35 for Mac) and IBM® SPSS® software (version 25 for Windows).

Categorical variables were described by number and percentage and continuous variables by mean, standard deviation, median, minimum and maximum. For test of differences between the two groups, Fisher’s exact test was used for dichotomous variables, the chi-square test for non-ordered categorical variables and the ANOVA test for continuous variables.

The prediction of complications during the study with baseline characteristic variables was performed by using logistic regression. Odds ratio (OR) and 95% confidence intervals (CIs) were presented from these analyses with associated *p* value. All tests were two-tailed and conducted at a 0.05 significance level.

### Ethical Statement

The displayed study was carried out with respect of high ethical standards. All the studies have been approved, when required, by the appropriate ethics committee and have, therefore, been performed in accordance and in conformity to the World Medical Association Declaration of Helsinki (June 1964) and sub-sequent amendments.

### Informed Consent

All patients signed an informed consent for the procedures. For this type of study, formal consent is not required.

## Results

**Fig. 4:**
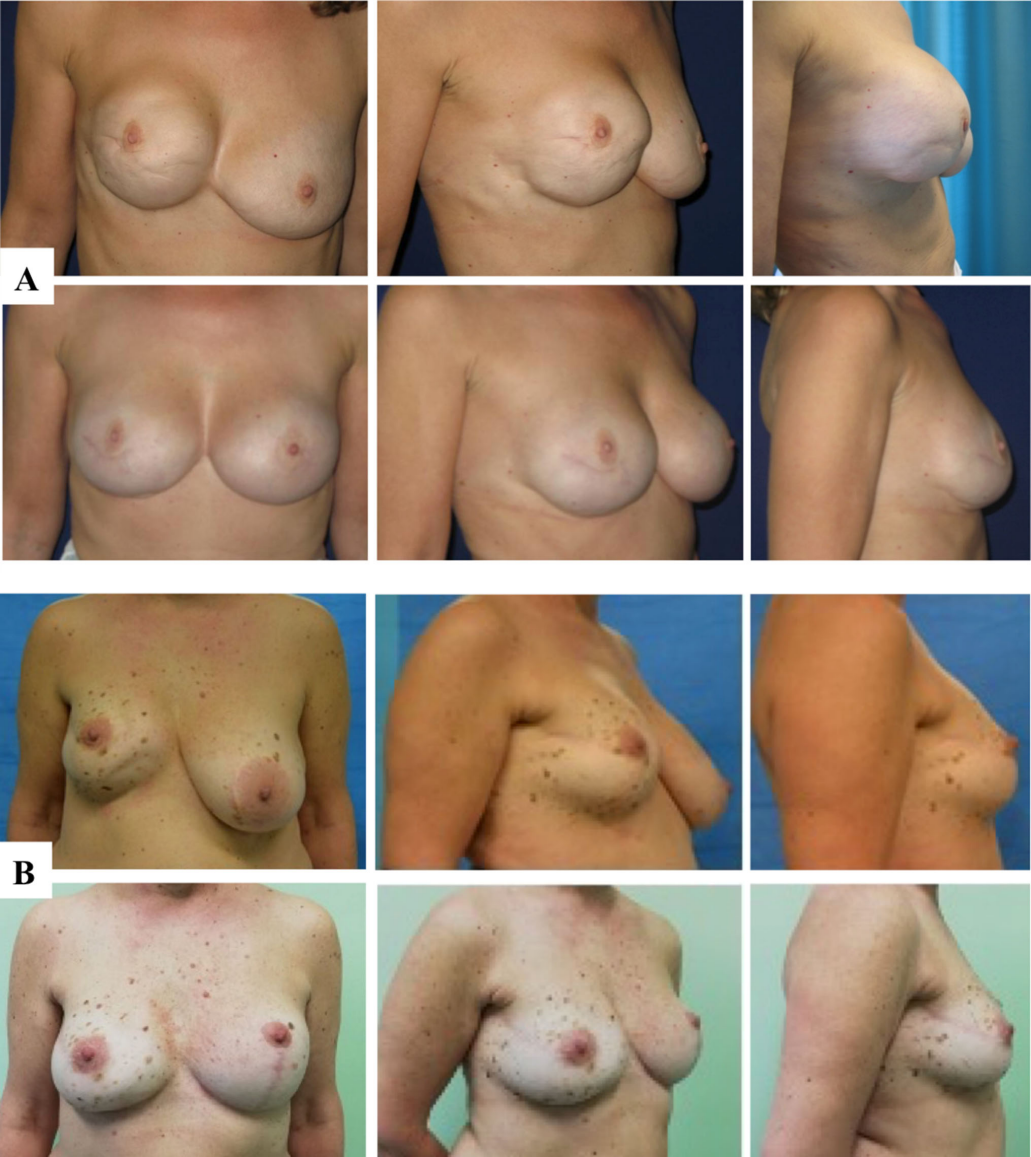
**A**, **B** Two clinical cases, pre-operative (above) and 1.3 years post-op (below) after breast exchange with complete implant wrapping with porcine-derived ADM and contralateral symmetrization

42 patients who underwent surgical treatment of CC affecting 46 breasts after reconstruction with breast implant were collected in the two groups (Control vs. ADM group). No differences were observed in patients’ demographics (Table 1), surgical details, post-operative management and follow-up data (Table 2) between the two groups. The mean follow-up time was 28 ± 3 months in the ADM group and 30 ± 4 months in the control group (Fig. 4).

**Table 1:** Description of the cohort: patient demographics and breast characteristics

		Case Group	Control Group	*p* value
Patients (*N*)		18	24	
Breasts (*N*)		22	24	
Age yo (mean ± SD)		57 ± 7 (range 45-72)	55 ± 8 (range 48-75)	0.917
BMI (mean ± SD)		24 ± 4 kg/m^2^ (range 18-32)	26 ± 4 kg/m^2^ (range 20-35)	0.066
Laterality for each patient	Monolateral ADM revisions for CC treatment	14 (77.8%)	24 (100%)	
	Bilateral surgery	4 (22.2%) ADM revisions for CC treatment	12 (50%) Controlateral symmetrization	
Weight distribution	Normal weight/overweight	16 (94.4%)	23 (96.68%)	0.609
	Obese	1 (5.6%)	3(12.5%)	
Comorbidities	Diabetes	1 (5.6%)	2 (8.33%)	0.533
	Rheumatic disease	4 (22.2%)	2 (8.33%)	0.045
	Hypertension	3 (16.7%)	6 (25%)	0.624
	Asthma	2 (11.1%)	1 (4.18%)	0.223
	Vascular diseases	4 (22.2%)	2 (8.33%)	0.008
Smoking habit	Current smokers	3 (16.7%)	7 (29.1%)	0.111
	Ex-smokers or no-smokers	19 (84.3%)	30 (70.9%)	
ASA scale	1	0	1 (4.18%)	0.522
	2	18 (100%)	23 (95.82%)	
	3	0		
Hx of chemotherapy		12 (66.7%)	14 (58.3%)	0.351
Hx of breast RT		15 (68.2%)	15 (68.2%)	0.065
Pinch test (on the breast flap) < 1cm		16 (72.7%)	9 (37.5%)	0.08
Previous devices for each breast	Primary expanders	6 (27.3%)	1 (4.18%)	0.377
	Primary implants	2 (9.1%)	6 (25%)	
	Secondary prosthesis	10 (45.5%)	14 (58.3%)	
	Tertiary prosthesis or more	4 (18.2%)	3 (12.5%)	

No statistical significant differences were observed between the two groups

**Table 2:** Surgery details and post-operative recovery

		ADM group	Control group	*p* value
Type of incision	Lateral	19 (86.4%)	23 (95.82%)	0.215
	Inverted T	1 (4.6%)	0	
	Periareolar	1 (4.6%)	0	
	inframammary	1 (4.6%)	1 (4.18%)	
Implant type	Medium height and moderate projection	12 (54.5%)	18 (75%)	0.053
	Low height and moderate plus projection	1 (4.6%)	4 (16.6%)	
	Medium height and moderate plus projection	9 (40.9%)	1 (4.18%)	
	Round moderate projection	0	1 (4.18%)	
Implant volume(mean ± sd)		363 ± 95cc	336 ± 106cc	0.387
Implant volume < 400cc		17 (77.3%)	14 (58.3%)	0.320
Operative time(mean ± sd)		90 ± 20 minutes	110 ± 51 minutes	0.120
Drainage removal < 7days		16 (82.7%)	17 (71.82%)	0.574

No statistical significant differences were observed between the two groups

### Capsular Contracture and Complications

**Fig. 5:**
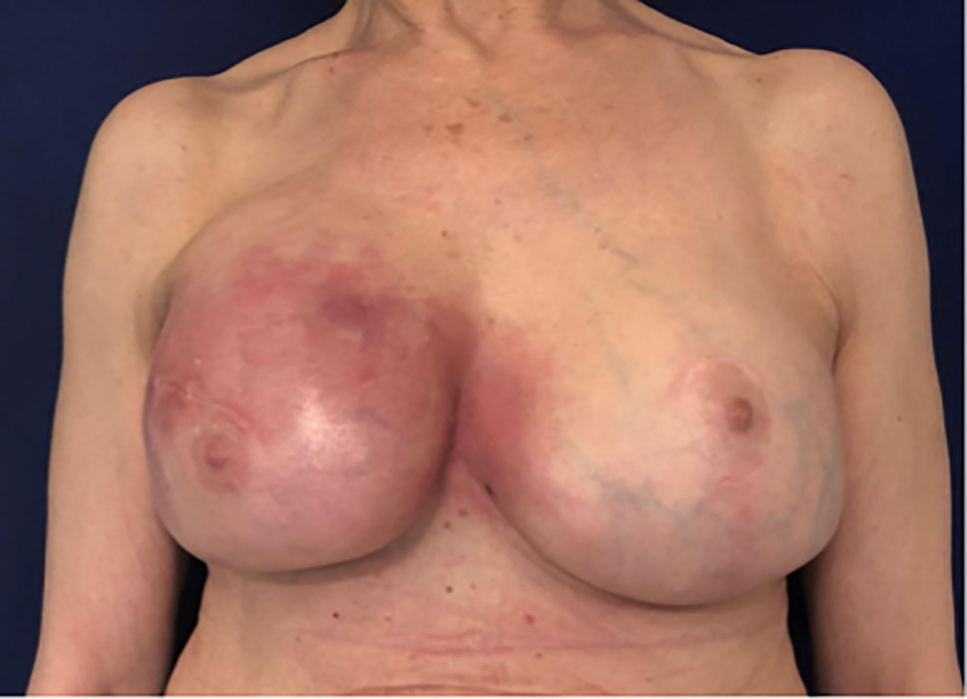
Clinical case of red breast syndrome

**Fig. 6:**
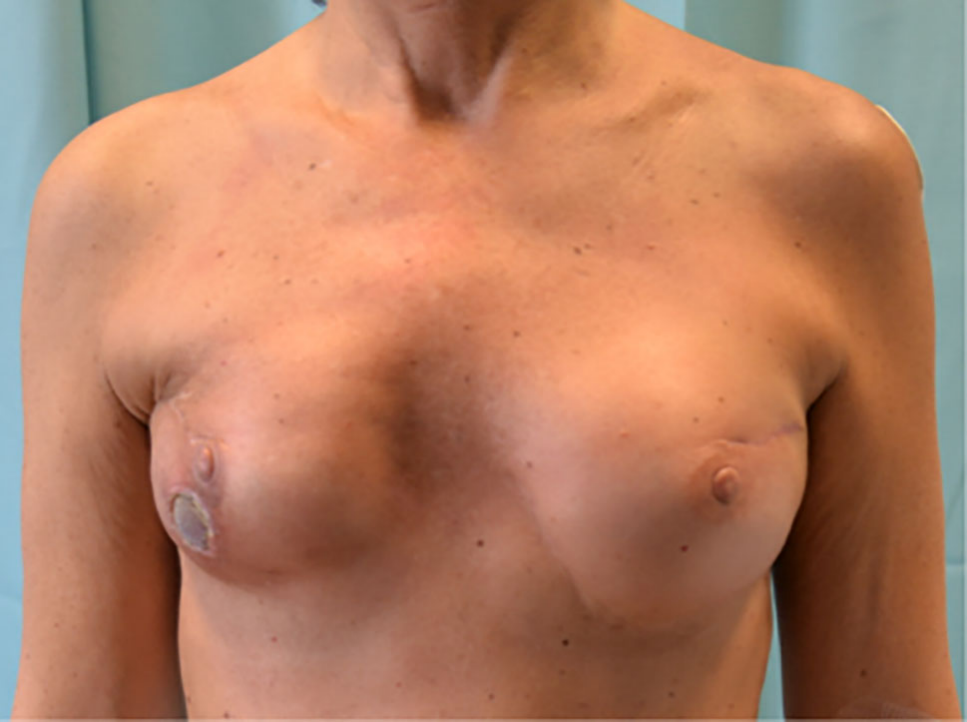
Clinical case of breast ulceration

**Fig. 7:**
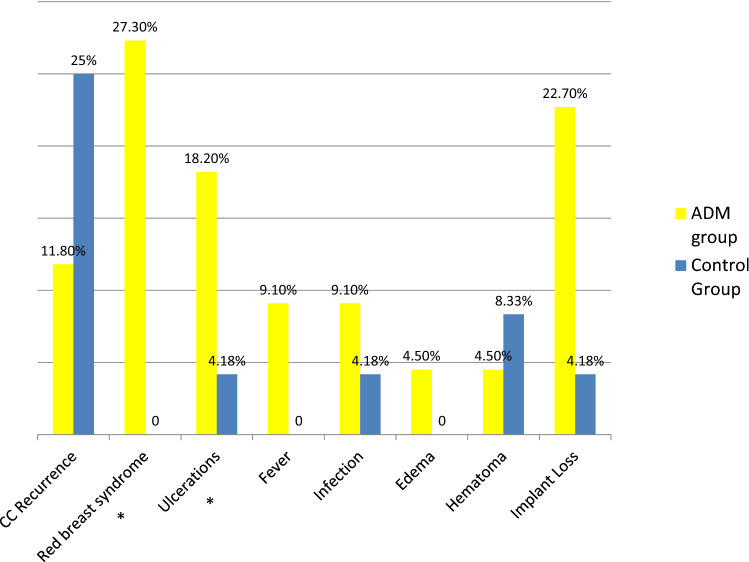
Graphical representation of CC recurrence rate and complications rate in the two groups. * statistical significant difference

The CC recurrence was lower in the ADM group (11.7%–2/17) compared to the control group (25 %–6/23) even if without statistical significance (*p* = 0.261). On the contrary, the rate of complication was higher in the ADM group. Implant losses were higher in the ADM group even if without statistical significance (22.7%–5/22 cases in the ADM group vs. 4.2%–1/24 in the control group, *p* = 0.075). On the other hand, a significant statistical difference was observed in the rate of minor complications such as red breast syndrome (Fig. 5) and skin ulceration rate (Figs. 6 and 7).

Univariable logistic regressions recognized previous radiation as a significant predictor for developing both CC recurrence and implant loss (OR 6, *p* = 0.046 and OR 18.667, *p* = 0.023, respectively) in both groups. In addition, the multivariate analysis including (age, BMI, smoke as other variables) revealed an increased odds ratio for CC recurrence in case of implant volume higher than 400 cc (OR = 118.968, *p* = 0.005) without differences in the two groups.

### Patient Satisfaction and Quality of Life

Patients belonging to different groups showed comparable values in all items before surgery which significantly differed in the post-operative Satisfaction of Breast Scale, (normality in clothes, size of the reconstructed breast, being able to wear clothes that are more fitted, how breasts are lined up in relation to each other, how comfortably bras fit, how equal in size are breast to each other) with a significantly better result in ADM group vs. the control group (56.17 ± 9.86 vs. 46.44 ± 6.0; *p* = 0.017).

As for intra-group differences before and after surgery, both groups showed improvement in all items. A statistical significant difference in the improvement of breast *Q* values, in favor of the ADM group, was observed in the Physical Well-Being of the Chest Scale (relief from thorax muscles pain, tension, discomfort on the breast, difficulties in arms moving) and in the Satisfaction of Breast Scale, (normality in clothes, size of the reconstructed breast, being able to wear clothes that are more fitted, how breasts are lined up in relation to each other, how comfortably bras fit, how equal in size are breast to each other) (Table 3A). On the contrary, the improvement in the Sexual Well-Being Scale (satisfaction of sexual life, comfort while having sex and sexual attractiveness without clothes) was higher in the control group (Table 3B).

**Table 3:** Breast Q reconstructive module scores (partial) paired sample *t* test value within the 2 groups

		M	SD	SE	*t*	*p*
*A. ADM group*						
PSY	Before	56.58	9.931	2.867	− .757	.465
	After	59.33	13.473	3.889		
SEX	Before	41.50	13.420	3.874	− 1.641	.129
	After	48.83	12.328	3.559		
CHEST	Before	41.33	25.134	7.255	− 2.883	.015*
	After	58.50	19.370	5.592		
BREAST	Before	41.50	10.892	3.144	− 4.709	.001*
	After	56.17	9.861	2.847		
*B. Control group*						
PSY	Before	55.00	11.192	3.731	− 1.818	.107
	after	58.00	11.683	3.894		
SEX	before	37.00	11.281	3.760	− 2.824	.022*
	after	47.11	11.537	3.846		
CHEST	before	52.33	16.718	5.573	1.349	.214
	after	47.67	16.971	5.657		
BREAST	before	39.22	9.985	3.328	− 1.818	.108
	after	46.44	6.002	2.001		

*PSY* Psychosocial Well-Being Scale, *SEX* Sexual Well-Being Scale, *CHEST* Physical Well-Being of the Chest Scale, *BREAST* Satisfaction of Breast Scale. When there is a significant result at *p* < 0.05, * is indicated. (M = mean, N = sample size, SD = standard deviation, SE = standard error, *t* = *t* value, *p* = *p* value) When there is a significant result at p<0.05, * is indicated

## Discussion

Capsular contracture is a problematic issue that has affected and is still affecting plastic surgery practice for a long time. Acellular dermal matrixes placed in various manners have been proposed to decrease the rate of CC recurrence. Clinical and patients’ reported outcomes were retrospectively compared in the present study in order to define the impact of a porcine-derived ADM in improving CC treatment. Anterior “complete” ADM implant coverage showed promising results in limiting capsular contracture recurrence and improving patients’ satisfaction in breast reconstruction patients [18]. As reported by Liu et al. [30], previous studies demonstrated the role of partial and anterior ADM coverage in reducing CC formation and recurrence both in breast reconstruction and breast augmentation [18, 19, 27, 31]. Indeed, we postulated that a complete isolation of the prosthesis also from the posterior capsule, left in place after anterior capsulectomy to reduce post-operative complications, could be beneficial to further reduce CC recurrence, improving patient well-being. A complete ADM coverage could limit the pathologic process of capsule formation tridimensionally [27].

Even if non-statistically significant, the results confirmed the role of ADM in reducing the risk capsular contracture development and recurrence, according to previous studies [18, 19]. A larger study could corroborate the significance of the results. Capsular contracture recurrence of grade III and IV showed significant relation with history of RT and implant volume greater than 400 cc. Both RT and high implant volume are known risk factors for CC recurrence [5, 30, 32]. Even though ADM application is considered a protective factor before adjuvant RT [26], its protective effects appeared limited when the reconstruction with ADM is performed after RT [33, 34].

Indeed, according to previous studies, the use of ADM was associated with higher rates of complication (ulceration and red breast syndrome) with significant correlation between RT and implant loss [35, 36]. No other factor alone showed association with implant loss in univariate analysis.

Our results confirmed that preoperative RT is a significant predictor of both implants loss and CC recurrence after surgical revision with and without ADM. Proper selection of patients and indication for autologous breast reconstruction should be considered in previously irradiated breasts [37].

As for patients reported outcome, the ADM improved Breast Q scores in tools such as general satisfaction, chest well-being and implants satisfaction when compared to breast implant exchange alone. A scarce literature dealing with Patient Perceived Outcome concerning revision procedures for CC specifically is available. Interestingly, BQ scores collected from patient undergoing breast implant revision for breast contour or asymmetry are similar to values collected in patients affected by CC [38]. According to previous study, ADM-assisted breast reconstruction is associated with high satisfaction rates when performed in a selected group of patients [39].

As for the enhanced post-operative results in the ADM group, they could be explained by the improved quality of soft tissue covering prosthesis anteriorly and with the application of a dermal matrix over the posterior capsule, left in place after anterior capsulectomy. In addition, an efficacious unloading of the weight of the prostheses over the suture of the matrix could explain the improved chest well-being score in the ADM group [28, 40]. On the contrary, the significant improvement of sexual well-being after surgery in the control group could be related to the differences in preoperative sexual well-being, which was lower in the control group.

The benefits in terms of improved patients’ reported outcome and protection against CC recurrence should be carefully weighed with the risk of complications and reconstructive failure and discussed with patients through a thorough discussion.

The main limitations of the present study include its retrospective nature, and the relative low number of patients involved. In addition, we report a clinical experience with a porcine-derived ADM and studies performed with different ADM should be performed. Moreover, the role of complete and partial coverage with ADM in preventing CC recurrence could be compared.

Further studies based on larger cohorts performing cost analysis should be done in long term to define the impact of different confounders in capsular contracture development and the effect of this technique in the overall costs of the procedure.

## Conclusion

Complete implant coverage by ADM may reduce the risk of CC recurrence in breast reconstruction. An accurate patient selection allows to minimize complications improving patient well-being and satisfaction.

Previous RT represents the main contraindication, as a risk factor for implant loss and capsular contracture recurrence. These results may represent the basis for future studies to determine an ideal flow chart to accomplish optimal outcomes in the treatment of CC and to achieve improved aesthetic and functional results breast reconstruction.

## References

[1] Rubio IT, Wyld L, Esgueva A, Kovacs T, Cardoso MJ, Leidenius M (2019). Variability in breast cancer surgery training across Europe: an ESSO-EUSOMA international survey. Eur J Surg Oncol.

[2] Tevlin R, Brazio P, Tran N, Nguyen D (2020) Immediate targeted nipple-areolar complex re-innervation: Improving outcomes in immediate autologous breast reconstruction. J Plast Reconstr Aesthet Surg 10.1016/j.bjps.2020.11.021 33341386

[3] Hakelius L, Ohlsén L (1992). A clinical comparison of the tendency to capsular contracture between smooth and textured gel-filled silicone mammary implants. Plast Reconstr Surg.

[4] Spear SL, Baker Jr JL (1995) Classification of capsular contracture after prosthetic breast reconstruction. Plast Reconstr Surg 96(5):1119–1123 (discussion 1124) 7568488

[5] Bachour Y, Bargon CA, de Blok CJM, Ket JCF, Ritt M, Niessen FB (2018). Risk factors for developing capsular contracture in women after breast implant surgery: a systematic review of the literature. J Plast Reconstr Aesthet Surg.

[6] Salzberg CA, Ashikari AY, Berry C, Hunsicker LM (2016). Acellular dermal matrix-assisted direct-to-implant breast reconstruction and capsular contracture: a 13-year experience. Plast Reconstr Surg.

[7] Namnoum JD, Moyer HR (2012). The role of acellular dermal matrix in the treatment of capsular contracture. Clin Plast Surg.

[8] Adams Jr WP (2009) Capsular contracture: what is it? What causes it? How can it be prevented and managed? Clin Plast Surg 36(1):119–126, vii 10.1016/j.cps.2008.08.007 19055967

[9] Araco A, Caruso R, Araco F, Overton J, Gravante G (2009). Capsular contractures: a systematic review. Plast Reconstr Surg.

[10] Barnsley GP, Sigurdson LJ, Barnsley SE (2006). Textured surface breast implants in the prevention of capsular contracture among breast augmentation patients: a meta-analysis of randomized controlled trials. Plast Reconstr Surg.

[11] El-Sheikh Y, Tutino R, Knight C, Farrokhyar F, Hynes N (2008). Incidence of capsular contracture in silicone versus saline cosmetic augmentation mammoplasty: a meta-analysis. Can J Plast Surg.

[12] Embrey M, Adams EE, Cunningham B, Peters W, Young VL, Carlo GL (1999). A review of the literature on the etiology of capsular contracture and a pilot study to determine the outcome of capsular contracture interventions. Aesthetic Plast Surg.

[13] Pajkos A, Deva AK, Vickery K, Cope C, Chang L, Cossart YE (2003). Detection of subclinical infection in significant breast implant capsules. Plast Reconstr Surg.

[14] Wong CH, Samuel M, Tan BK, Song C (2006). Capsular contracture in subglandular breast augmentation with textured versus smooth breast implants: a systematic review. Plast Reconstr Surg.

[15] Ganon S, Morinet S, Serror K, Mimoun M, Chaouat M, Boccara D (2021). Epidemiology and prevention of breast prosthesis capsular contracture recurrence. Aesthetic Plast Surg.

[16] Chong SJ, Deva AK (2015). Understanding the etiology and prevention of capsular contracture: translating science into practice. Clin Plast Surg.

[17] Lam MC, Walgenbach-Brünagel G, Pryalukhin A, Vorhold J, Pech T, Kalff JC (2019). Management of capsular contracture in cases of silicone gel breast implant rupture with use of pulse lavage and open capsulotomy. Aesthetic Plast Surg.

[18] Cheng A, Lakhiani C, Saint-Cyr M (2013). Treatment of capsular contracture using complete implant coverage by acellular dermal matrix: a novel technique. Plast Reconstr Surg.

[19] Wagner DS, Mirhaidari SJ (2021). Capsulectomy, implant exchange, and placement of acellular dermal matrix is effective in treating capsular contracture in breast augmentation patients. Aesthet Surg J.

[20] Baker Jr JL, Chandler ML, LeVier RR (1981) Occurrence and activity of myofibroblasts in human capsular tissue surrounding mammary implants. Plast Reconstr Surg 68(6):905–912 10.1097/00006534-198112000-00010 7301985

[21] Basu CB, Leong M, Hicks MJ (2010). Acellular cadaveric dermis decreases the inflammatory response in capsule formation in reconstructive breast surgery. Plast Reconstr Surg.

[22] Ksander GA, Vistnes LM (1985). The incidence of experimental contracture varies with the source of the prosthesis. Plast Reconstr Surg.

[23] Yu D, Hanna KR, LeGallo RD, Drake DB (2016). Comparison of histological characteristics of acellular dermal matrix capsules to surrounding breast capsules in acellular dermal matrix-assisted breast reconstruction. Ann Plast Surg.

[24] Stump A, Holton LH, Connor J, Harper JR, Slezak S, Silverman RP (2009). The use of acellular dermal matrix to prevent capsule formation around implants in a primate model. Plast Reconstr Surg.

[25] Komorowska-Timek E, Oberg KC, Timek TA, Gridley DS, Miles DAG (2009). The effect of AlloDerm envelopes on periprosthetic capsule formation with and without radiation. Plast Reconstr Surg.

[26] Kim A, Jung JH, Choi YL, Pyon JK (2019). Capsule biopsy of acellular dermal matrix (ADM) to predict future capsular contracture in two-stage prosthetic breast reconstruction. J Plast Reconstr Aesthet Surg.

[27] Leong M, Basu CB, Hicks MJ (2015). Further evidence that human acellular dermal matrix decreases inflammatory markers of capsule formation in implant-based breast reconstruction. Aesthet Surg J.

[28] Bassetto F, Pandis L (2020). Clinical experience with Surgimend in breast reconstruction: an overview. Br J Hosp Med (Lond).

[29] Stolpner I, Heil J, Feißt M, Karsten MM, Weber WP, Blohmer JU (2019). Clinical validation of the BREAST-Q breast-conserving therapy module. Ann Surg Oncol.

[30] Liu J, Hou J, Li Z, Wang B, Sun J (2020). Efficacy of acellular dermal matrix in capsular contracture of implant-based breast reconstruction: a single-arm meta-analysis. Aesthetic Plast Surg.

[31] Spear SL, Sher SR, Al-Attar A, Pittman T (2014). Applications of acellular dermal matrix in revision breast reconstruction surgery. Plast Reconstr Surg.

[32] Lee KT, Mun GH (2015). Prosthetic breast reconstruction in previously irradiated breasts: a meta-analysis. J Surg Oncol.

[33] Parks JW, Hammond SE, Walsh WA, Adams RL, Chandler RG, Luce EA (2012). Human acellular dermis versus no acellular dermis in tissue expansion breast reconstruction. Plast Reconstr Surg.

[34] Valdatta L, Cattaneo AG, Pellegatta I, Scamoni S, Minuti A, Cherubino M (2014) Acellular dermal matrices and radiotherapy in breast reconstruction: a systematic review and meta-analysis of the literature. Plast Surg Int 2014:472604 10.1155/2014/472604 PMC4055390 24987526

[35] Negenborn VL, Young-Afat DA, Dikmans REG, Smit JM, Winters HAH, Don Griot JPW et al (2018) Quality of life and patient satisfaction after one-stage implant-based breast reconstruction with an acellular dermal matrix versus two-stage breast reconstruction (BRIOS): primary outcome of a randomised, controlled trial. Lancet Oncol 19(9):1205–1214 10.1016/S1470-2045(18)30378-4 30104147

[36] Wagner RD, Braun TL, Zhu H, Winocour S (2019). A systematic review of complications in prepectoral breast reconstruction. J Plast Reconstr Aesthet Surg.

[37] Khajuria A, Charles WN, Prokopenko M, Beswick A, Pusic AL, Mosahebi A (2020). Immediate and delayed autologous abdominal microvascular flap breast reconstruction in patients receiving adjuvant, neoadjuvant or no radiotherapy: a meta-analysis of clinical and quality-of-life outcomes. BJS Open.

[38] Rosson GD, Shridharani SM, Magarakis M, Manahan MA, Basdag B, Gilson MM (2013). Quality of life before reconstructive breast surgery: a preoperative comparison of patients with immediate, delayed, and major revision reconstruction. Microsurgery.

[39] Negenborn VL, Dikmans REG, Bouman MB, Wilschut JA, Mullender MG, Salzberg CA (2018) Patient-reported Outcomes after ADM-assisted Implant-based breast reconstruction: a cross-sectional study. Plast Reconstr Surg Glob Open 6(2):e1654 10.1097/GOX.0000000000001654 PMC5865927 29616167

[40] Gui G, Tsang FJ (2019) Meshed enhanced Hammock’ or ‘Tent’: A new patient centred pre-pectoral one-stage immediate breast reconstruction technique for varying ptosis. Ann Plast Reconstr Surg 3(1):1026

